# Proceedings from the CIH^LMU^ 2025 symposium: the role of artificial intelligence in health systems strengthening to achieve One Health

**DOI:** 10.1186/s12919-026-00377-1

**Published:** 2026-04-29

**Authors:** Martha Chipinduro, Patrícia Ramgi, Julieth Lalashowi, Lalem Belay, Surafel Worku Megersa, Temesgen Chala, Dawit Ejigu, Eyob Abera, Shambhunath Mohan, Michael Montgomery, Lidia Veliaminova, Given Chiumya, Adea Borova, Vahuka Valiyakath, Guenter Froeschl

**Affiliations:** 1 Teaching & Training Unit, Institute of Infectious Diseases and Tropical Medicine, University Hospital, LMU, Munich, Germany; 2 Center for International Health, Ludwig-Maximilians-Universität, Munich, Germany; 3 The Health Research Unit-Zimbabwe, Biomedical Research and Training Institute, Harare, Zimbabwe; 4 Faculty of Medicine and Health Sciences, Midlands State University, Gweru, Zimbabwe; 5 Instituto Nacional de Saúde, Marracuene, Mozambique; 6 Department of Psychiatry, Saint Paul’s Hospital Millennium Medical College, Addis Ababa, Ethiopia

**Keywords:** Artificial intelligence, One health, Health system strengthening, Digital health, Student initiative

## Abstract

The development of Artificial Intelligence (AI) is rapidly advancing, and AI tools are being integrated into many aspects of daily life, including medical care and public health. The full extent to which AI related tools can be implemented in order to strengthen health systems are a matter of continued debate. The Center for International Health at Ludwig-Maximilians-Universität’s (CIH^LMU^) 2025 student-led symposium on "*The Role of Artificial Intelligence in Health Systems Strengthening to Achieve One Health*" deliberated on the transformative potential of AI in global health. The primary focus of the symposium was to explore how AI can be leveraged to strengthen health systems. Presentations were delivered by health experts on AI from Africa and Europe. The event provided a platform for experts and students to discuss how AI can be harnessed to improve healthcare delivery, disease surveillance, and research. Discussions resonated around AI health-related research, responsible AI integration, advocacy for AI-related policies, and challenges associated with AI integration in health, such as data privacy and the need for robust governance frameworks. Emphasis was placed on the importance of context-specific implementation, interdisciplinary collaboration, and robust governance to ensure AI’s responsible integration into One Health approaches. The symposium concluded that AI holds immense promise to strengthen health systems by improving efficiency, equity, and responsiveness. However, ethical, infrastructural, and regulatory challenges must be addressed, particularly in low- and middle-income countries (LMICs).

## Introduction

Artificial Intelligence (AI) has the potential to transform medical care and public health systems, with rapid advances having occurred over the past several years. This is particularly relevant in low- and middle-income countries (LMICs) where there is significant shortage and unequitable distribution and access to health care services [1]. AI can help foster health services by assisting in diagnosis, treatment planning, and clinical triage [2]. However, implementing AI tools presents significant difficulties, particularly in LMICs, due to weak infrastructure and limited digital capacity [3]. The Center for International Health of the Ludwig-Maximilians-Universität (CIH^LMU^) 2025 Symposium, titled “*The Role of Artificial Intelligence in Health Systems Strengthening to Achieve One Health*,” held on March 21, 2025, was organized to provide a platform for discussion on the use of AI in health. In particular, presentations and discussions focused on current applications, existing challenges and solutions for sustainable AI implementation in health systems strengthening.

## Methods

### Symposium organization

Since 2012, CIH^LMU^ has hosted annual student-led *Symposia Series on Global Health* as part of the PhD Medical Research–International Health (MR-IH) and MSc International Health (IH) programs. The 2025 symposium was organized by 12 students, supported by two program coordinators and head of the teaching and training unit, and was preceded by workshops on project management, communication and conflict management. The symposium, the 19th in this series, gave the students the opportunity to organise a scientific event of international importance and to collaborate with professional experts on AI. Students acquired three European Credit Transfer System points for organising a successful symposium.

### Topic selection

All students participated in identifying symposium topics related to One Health, in order to align with the global approach to health. The students agreed on AI as the primary focus of the symposium with health services or health systems as a secondary focus. A final consensus was made on; “*The Role of Artificial Intelligence in Strengthening Health Systems to Achieve One Health*”. The students reviewed available literature on the topic in a scoping manner and defined four priority areas for inclusion: 1. AI for optimizing health system operations and resource allocation in low resource settings; 2. Applying AI for improving efficiency in clinical research; 3. Ethical and regulatory considerations in the use of AI in healthcare; 4. Leveraging AI for animal/human disease surveillance, environmental surveillance and early warning systems. All students proposed potential speakers based on online search, suggestions by supervisors, and consultations with their respective personal networks, and email invitations were sent. Ultimately, eight speakers agreed to participate in the symposium.

### Symposium delivery

The CIH^LMU^ student-led symposium was conducted in person from 2012 until 2020. Since 2021, due to the COVID-19 pandemic, the event was shifted to a hybrid format with online participation facilitated by Zoom Meetings (Zoom Video Communications, Inc, San Jose, California, USA). In-presence participants were invited to the Main Lecture Hall of the LMU University Hospital at the Inner City Campus. Registration ran from December 17, 2024 until March 21, 2025. Each presentation lasted 30 min, followed by a 15-min discussion. The symposium had a total number of 175 online participants and 30 in person attendees. Symposium attendees holding a medical license were eligible for 9 Continuous Medical Education credit points.

### Technical sessions

The technical sessions are presented through brief speaker biographies followed by a summary of the presentation overview and key discussion points.

### Keynote presentation: “Many AIs, One Health”—Björn Schuller,

#### Biography

Björn Schuller is a professor at the Technische Universität München (TUM) and Imperial College, London. He is chair of Health Informatics at TUM, Germany and co-founding Chief Executive Officer and current Chief Security Officer of audEERING Gmbh. He is a fellow of the Association for Computing Machinery, field Chief Editor of *Frontiers in Digital Health* and Editor-in-Chief of the *AI Open Journal*.

#### Overview

The key note highlighted AI as a broad and rapidly evolving field with applications across diagnosis, treatment and maintenance/rehabilitation. Using examples of audio and visual AI sensors, the presentation demonstrated how these could be leveraged for human, animal and environmental health. AI acoustic sensors placed on particular body parts can track sounds (“Voices of the body”), for example, breath movements, pulse [4]. Wearable devices gather physiological data such as blood sugar levels and blood pressure whilst videos provide data on facial expression, habit expression such as nail biting and emotions [5]. Machine learning on these data results in AI models which can be used for screening, diagnosis and treatment monitoring of various disorders such as, mental health, musculoskeletal, neurocognitive and respiratory disorders among many. AI sensor application in animal health have included mice autism detection via ultrasound vocalization and in passive acoustic monitoring. Visualisation of environments along with changing soundscapes have been used to highlight the impact of human disturbances on the ecosystem and its influences on human mental health [6].

#### Discussion and conclusion

Future research should focus on how AI can support environmental and policy interventions that promote mental wellbeing, such as reducing noise pollution and restoring natural environments. Such interventions may include creating and reinforcing positive sounds (bird and water sounds, etc.), restoring and maintaining vegetation and policy changes, such as, ban on heavy vehicle movements in residential places at night. The long-term vision is the development of interconnected AI bio-surveillance systems supporting human, animal, and environmental health.

Key challenges include data privacy and security, computational demands, interoperability limitations, and ensuring reliability of AI models across diverse environments. Furthermore, there is lack of policies on data sharing that promote equity, fairness and trust to access and use of AI generated data. Overall, AI has the potential to produce interconnected multidisciplinary systems based on large-scale One Health models for global bio-surveillance.

### “Artificial Intelligence and Digital Precision Health (10P Health): The 21 st Century Health and Wellness Paradigm”- Georgi Chaltikyan,

#### Biography

Georgi Chaltikyan is a professor and the Head of Digital Health at the European Campus Rottal-Inn, Deggendorf Institute of Technology. He is the founder and Head of Research at the Education Center of Digital Head at Russian-Armenian University, and the Founder and Chairman of the Armenian Association of Digital Health. He has extensive experience in healthcare education, research, and management and has been a World Health Organization (WHO) Digital Consultant since 2019.

#### Overview

The presentation explored the rapid growth of digital health technologies, including telemedicine, robotics, health analytics, and precision medicine. Mobile health applications have become more prevalent with nearly 60% of smartphone users using some form of a digital health care application and nearly 350,000 digital health applications being available [7, 8]. Geoffrey Hinton, known as the "Godfather of AI” noted that healthcare has the most promising revolutionizing application of AI. For instance, robotics in healthcare introduced a paradigm shift to medical education and training, and surgery.

Advances in cloud computing, large datasets, language learning models, and deep learning have accelerated the development of AI applications ranging from clinical decision support to drug development and patient self-management. TxGemma AI (predicts the safety and efficacy of potential new treatments) and Pixel Watch 3 (for loss of pulse detection) are examples. Concepts such as Digital Precision Health (10P Health) and digital twins were presented as emerging models integrating predictive, preventive, personalized, and participatory healthcare.

However, lack of evaluation benchmarks and metrics, domain data limitations and behavior alignment are barriers. Data privacy and security, fairness and bias, ethical regulatory and economic considerations remain considerable challenges to be overcome.

#### Discussion and conclusion

AI-enabled precision medicine can support One Health by expanding its traditional focus on individualized human care toward an integrated systems approach that combines human, animal, pathogen, and environmental data to improve prevention, surveillance, and response across interconnected health domains. By integrating genomic data from humans, livestock, wildlife, and environmental sources, AI can help identify cross-species transmission pathways and emerging zoonotic threats while strengthening antimicrobial resistance tracking across clinical, veterinary, agricultural, and environmental sectors to support more responsible antimicrobial stewardship. At the same time, incorporating environmental exposure data such as climate variables, pollution, water quality, and land-use changes allows AI to better predict disease risks shaped by ecological conditions. Precision medicine can also link human health to food systems and biodiversity by integrating nutrition, microbiome, and agricultural data to promote healthier and more sustainable production systems. Together, these capabilities enable a shift from reactive clinical care toward precision public health and precision ecosystem surveillance, where AI supports early warning systems, targeted interventions, and sustainable health policies that simultaneously protect human wellbeing, animal health, and environmental integrity.

### “Use of AI platforms (AfyaData) to strength the health system using the One Health approach”—Inocêncio Chongo

#### Biography

Inocêncio Chongo holds a Veterinary Medicine Degree from Eduardo Mondlane University, Mozambique, a Master’s Degree in Laboratory Epidemiology, and is a PhD candidate in Tropical Diseases and Global Health at Nova University of Lisbon, Portugal. He is working at the National Institute of Health of Mozambique as a Researcher of Zoonotic, (Re)emerging and Neglected Diseases and is the coordinator of the One Health Platform of Mozambique and the One Health Approach Focal Point in Mozambique for Africa Centres for Disease Control and Prevention.

#### Overview

AfyaData is an open-source digital disease surveillance tool developed by the Southern African Centre for Infectious Disease Surveillance at Sokoine University of Agriculture, Tanzania. It enables real-time data collection and feedback mechanisms to enhance early detection and response to infectious diseases at the community level and can be used online and offline [9]. This tool was implemented in Mozambique, a low-income country suffering from natural disasters (droughts, floods and tropical cyclones), the consequences of climate change [10]. The country has an estimated population of 30 million, with an average of three doctors per 100,000 individuals. Half of Mozambican people live at least within one-hour walking distance to health facilities and emergence of zoonotic diseases such as rabies, zoonotic tuberculosis, salmonellosis, trypanosomiasis, brucellosis zoonotic avian flu and Crimean-Congo hemorrhagic fever is commonly experienced in the country [11]. AfyaData is accessible from any smartphone, from the community level up to the most specialized health facility. Health and environmental information entered on AfyaData is automatically processed and visualized in graphs, tables, and other formats. In Mozambique, the information is sent to the One Health Knowledge Repository and performs more complex analysis. The processed data is made available almost in real time allowing policy makers to make prompt decisions regarding emergent diseases or outbreaks.

AfyaData was initially used during the COVID-19 pandemic at Chókwè District in Mozambique. After the COVID-19 pandemic, the application continues to support surveillance of diseases, such as, Marburg Disease, Crimean-Congo Hemorrhagic Fever and Rift Valley Fever, throughout the country.

#### Discussion and conclusion

AfyaData demonstrates how community-based AI tools can strengthen surveillance and response systems in low-resource settings. Community engagement ensures responsible stewardship of One Health by all. Major challenges encountered with the use of the AfyaData platform included, limited power supply in some parts of Mozambique, limited community digital literacy, and the need for sustainable funding. The platform highlights the importance of simple, scalable AI-enabled tools for strengthening health systems through a One Health approach.

### “Diagnostics in the era of artificial intelligence: How do we still safeguard biosecurity?”—Talkmore Maruta,

#### Biography

Talkmore Maruta serves as the Director of Programs at the African Society for Laboratory Medicine. He holds a PhD in public health. He has more than 20 years of experience in the areas of laboratory systems strengthening and emergency preparedness and response. Dr Maruta has vast experience in biosafety and biosecurity.

#### Overview

AI is improving laboratory diagnostics though through automation, workflow optimization, and improved data interpretation [12]. While AI offers numerous advantages, concerns regarding data integrity, transparency, cybersecurity, workforce displacements, and ethical governance remain critical. Addressing these challenges requires a balance between technological advancement and stringent biosecurity measures. Strengthened regulatory frameworks and international collaboration are needed to ensure safe implementation.

The implementation of AI in healthcare must therefore be accompanied by policies that promote transparency, security, and accountability.

#### Discussion and conclusion

One Health initiatives rely on integrated datasets that combine human clinical data, veterinary, environmental, climate and ecological data. Secure integration of AI within One Health requires protecting interconnected human, animal, and environmental data systems. Biosecurity frameworks must address cyber-biosecurity risks, dual-use research concerns, and governance of biological data. Responsible AI integration to achieve One Health while safeguarding biosecurity requires strong policies, regular risk assessments, and cross-sector collaboration.

### “One Youth, One Health – perspectives and challenges to enhance mental health in the young using digital technology”—Johanna Löchner,

#### Biography

Johanna Löchner is a professor of Clinical Psychology and Psychotherapy for Children and Adolescents at the Friedrich-Alexander-University Erlangen Nürnberg. As a licensed psychotherapist for cognitive behavioral therapy, she has been treating children, adolescents, and adults for over 10 years and is an associated member of the German Center for Mental Health.

#### Overview

The presentation highlighted growing mental health challenges among youth, particularly following COVID-19, and the potential of digital tools to expand access to care. Childhood mental wellbeing represents an important predictor of adult mental health. The post-Covid era presents a vulnerable period for youths, with increasing prevalence of poor mental health, low treatment supply, and more treatment barriers. AI-enabled mental health technologies include tele-psychotherapy, chatbots, wearable monitoring, virtual reality interventions, and mobile health applications, with more than 50,000 existing mental mobile health applications [13].

The application of AI in psychotherapy has been explored in prevention, screening, diagnostics, therapy and aftercare. Among youths, AI-driven digital platforms have been used to support monitoring mental well-being, and to detect early signs of depression and anxiety [14].

Evidence suggests that internet-based cognitive behavioral therapy can achieve outcomes comparable to face-to-face therapy for psycho-somatic disorders, while blended approaches combining digital tools with human support show improved adherence and outcomes [15]. Additional evidence includes increased mental health literacy at six months of using the Mental Health Guide, an app-based intervention developed by lived experience experts (people who have lived with mental health conditions). Blended counselling (use of an application plus counselling by midwives**)** for young parents-to-be with psychosocial burden is another model that has shown promising results in reducing stress.

#### Discussion and conclusion

Despite promising evidence, gaps remain in adolescent high-risk populations (e.g. individuals with suicidal tendencies), and in many LMICs. High drop-out rates, limited log-term follow –up data, and ethical questions about AI-based therapeutic relationships remain concerns. Whether AI-supported psychotherapy aligns with key conventional psychotherapy principles (alliance, empathy, expectations, cultural background and competence) remains an ethical question. Strong legal and ethical frameworks, data protection, transparency, and quality assurance are essential for responsible implementation.

### “AI in biomedical imaging—from image quality to unsupervised anomaly detection” Julia Schnabel

#### Biography

Julia Schnabel is a professor for Computational Imaging and AI in Medicine at TUM, Germany. She is recognized with the TUM Liesel Beckmann Distinguished Professorship. She serves as the Director at the Institute of Machine Learning in Biomedical Imaging, Helmholtz Munich, Germany.

#### Overview

AI in biomedical imaging is transforming diagnostics through improved image acquisition, reconstruction, and automated anomaly detection [16]. Advances in self-supervised learning enable detection of imaging artifacts and identification of abnormalities without extensive labeled datasets, addressing radiologist workload challenges. Establishing models with sufficient validity that allow distinguishing physiological from pathological findings can be challenging, The possibility of detecting anomalies independently (unsupervised, without needing extensive expert input), paved the way for AI systems to identify deviations from normal anatomy and support automated clinical decision. Interestingly, AI using unsupervised anomaly detection coupled with language models can already be used to respond to clinicians’ questions regarding pathological images and the resulting answer used to rule-in or rule-out certain diagnoses.

#### Discussion and conclusion

AI imaging applications could extend to zoonotic disease surveillance, population health monitoring, and environmental health analysis. AI has the potential to enable analysis of very large imaging on geography and across populations for identification of geographic disease patterns and monitoring of environmental health impacts. However, risks include misclassification, over-normalization of abnormalities, and validation challenges. Despite these risks, AI shows strong potential to improve diagnostic efficiency and disease surveillance.

### “The role of artificial intelligence in maternal health”—Leyla Larsson,

#### Biography

Leyla Larsson is a public health professional and epidemiologist with a background in biomedical engineering and medical device design. She holds a Master's Degree in Reproductive and Sexual Health and is finalizing a PhD in Epidemiology and Public Health at LMU. Currently, she holds a post as a Research Fellow at the Clinical Research Department at the London School of Hygiene and Tropical Medicine where she coordinates a large trial investigating the impact of screening and treating for sexually transmitted infections during pregnancy and adverse maternal and neonatal outcomes.

#### Overview

Maternal mortality refers to deaths resulting from complications associated with pregnancy or childbirth. In 2020, nearly 800 women died each day from preventable pregnancy- and childbirth-related causes [17]. Approximately 95% of these deaths occurred in LMICs, particularly in sub-Saharan Africa. The primary causes of maternal deaths in this region include obstetric hemorrhage and hypertensive disorders. AI applications in maternal health include predictive risk modelling, remote monitoring, and digital decision support tools aimed at reducing maternal mortality and premature births, particularly in low-resource settings where most maternal deaths occur [18]. AI has been used to predict complications such as preterm birth, gestational diabetes, and pre-eclampsia [19, 20]. Remote monitoring is supported through AI tools like chatbots and contributes to optimizing health systems and promoting healthcare equity. For example, the Wysa, a chatbot for remote monitoring of mothers to enhance health information and help with the early detection of postnatal depression, allowing for intervention. Mothers who were highly engaged with the application saw a 12.7% reduction in depressive symptoms [21].

#### Discussion and conclusion

Ethical considerations in the use of AI tools for maternal health include data security, patient privacy and autonomy, and biases within AI models that can compromise data representativeness. Practical implementation challenges such as cost, the need for specialized training, and infrastructure limitations were highlighted. Future directions include integration of AI into national maternal health programs and the advancement of AI-driven clinical trials and personalized medicine which combine clinical, imaging, and genomic data. Responsible implementation and interdisciplinary collaboration are essential for impact.

### “AI in health research in Mozambique—are the ethics committees prepared?”—Esperança Sevene, Universidade Eduardo Mondlane

#### Biography

Esperança Sevene holds a Medical Doctors’ degree from Eduardo Mondlane University, Mozambique, a Master's in Pharmaco-epidemiology at the Autonomous University of Barcelona and a PhD in Medicine at the University of Barcelona. She is an Associate Professor of Clinical Pharmacology, Senior Researcher at the Manhiça Health Research Center and President of the National Bioethics Committee for Health. She is a member of the Africa-Europe Foundation Health Strategic Group, Maternal Immunization Working Group and the WHO Advisory Committee on Safety of Medicinal Products.

#### Overview

The presentation examined the readiness of ethics committees to review AI-related research, using Mozambique as a case study. The National Ethics Committee of Mozambique was formerly established in 2023 following the need for an ethical committee operating according to international standards to regulate malaria clinical trials. The committee has 13 members and a chair, who all cover their position on a non-remunerated voluntary basis. The committee receives a growing number of protocols in both Portuguese and English, along with a trend of increasing complexity of protocols applying AI based methodologies. On the other hand, the meetings to review the protocols are still being held in person and using hard copy documents. AI has only been introduced to support administrative screening processes, improving efficiency by identifying incomplete submissions and ethical risks.

However, AI cannot fully capture contextual cultural, linguistic and ethical considerations making human review essential.

On the other hand, the ethics committees receive protocols where AI was used by the investigators as a tool to write different parts of the document or that includes the use of AI in different study procedures. The ethics committee in Mozambique evaluates these protocols, always looking for three basic principles: justice, beneficence and autonomy. However, there remains a knowledge gap in understanding the full spectrum of ethical considerations where AI technologies are applied in research.

Ethics Committees in Mozambique and other low-income countries need to be prepared to deal with AI. Barriers to using AI in low-resource settings include the lack of internet and the lack of training of people on the technologies. The use of these tools might increase inequities as developing countries are far behind in preparedness.

#### Discussion and conclusion

AI can improve efficiency of ethics review processes but requires clear regulatory guidance. Key needs include training ethics committees, developing AI governance guidelines, and strengthening digital infrastructure. Without adequate guidance and understanding of the scope of AI related ethical considerations, the progressive adoption of AI technologies in research risks compromising principles of justice, beneficence and autonomy.

### Lessons learnt: students’ perspective on organising the symposium

Organising the symposium provided a valuable experiential learning opportunity that extended beyond academic requirements. Through pre-symposium training sessions, students learned the importance of working within a structured team, which strengthened coordination, decision-making, and problem-solving skills. Clear role allocation ensured accountability while also creating space for innovation and initiative. The training further promoted professional communication and supported effective conflict resolution.

A key priority was engaging speakers from both low- and high-resource settings, which provided important insights into contextual realities in integrating AI into One Health approaches. These interactions also fostered South–North dialogue and highlighted the importance of equitable global collaborations.

Importantly, the experience helped students see themselves as emerging professionals capable of contributing to international health discussions by convening experts in AI and One Health. Overall, organising the symposium empowered students with stronger leadership, communication, and networking skills essential for their future careers.

## Conclusion

The CIH^LMU^ 2025 Symposium provided a platform to more than 200 participants to explore how AI can be optimally leveraged to strengthen health systems towards achieving One Health. Presentations highlighted AI’s transformative potential across diagnostics, surveillance, maternal and mental health, imaging, and research ethics. Discussions emphasized that successful integration of AI depends on context specific implementation, data integrity, equitable access, strong governance and adequate technical infrastructure – particularly in low –resource settings where challenges remain significant. While many sessions focussed on the future potential of AI in One health, AfyaData provided a practical example of how AI can successfully integrate human, animal, and environmental health in a resource-limited context.

The symposium concluded that advancing AI for One Health requires addressing structural inequalities that shape health outcomes and access to technology. Key justice priorities include fostering equitable partnerships, ensuring fair representation in datasets and algorithms, developing AI solutions suitable for low-resource settings, and safeguarding biodiversity and ecosystems. Beyond knowledge exchange, the symposium was not only a platform for knowledge exchange, the symposium served as a call to action for the global health community to guide the responsible development of AI in health through cross-sector collaboration, continuous learning, and policies grounded in safety, inclusiveness, and transparency.

## Data Availability

Presentations from symposium speakers are available upon reasonable request and upon agreement by the respective presenters.

## Abbreviations

AI: Artificial Intelligence
BMZ: German Ministry for Economic Cooperation and Development
CIH^LMU^: Center for International Health at Ludwig-Maximilians-Universität
DAAD: German Academic Exchange Services
LLMs: Large Language Models
LMICs: Low- and Middle-Income Countries
LMU: Ludwig-Maximilians-Universität
MSc IH: MSc International Health
PhD MR-IH: PhD Medical Research-International Health
TUM: Technische Universität München
WHO: World Health Organization
